# Supplementary figures and images for: The synergistic effect of concatenation in phylogenomics: the case in Pantoea
Source: PeerJ. 2019 Apr 16;7:e6698. doi: 10.7717/peerj.6698 (PMC6474361; doi:10.7717/peerj.6698)

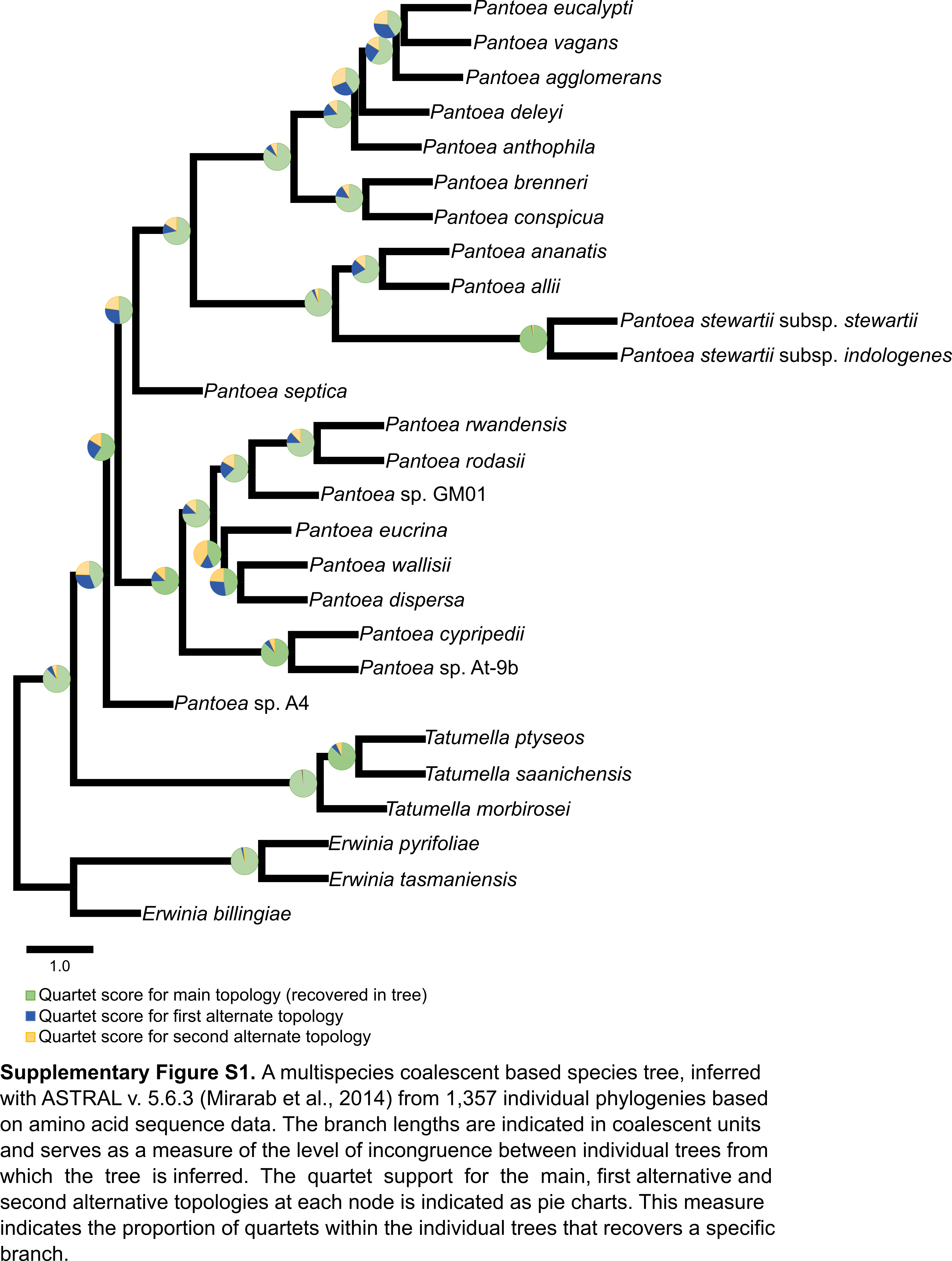

Supplement: Figure S1 [file peerj-07-6698-s001.png]

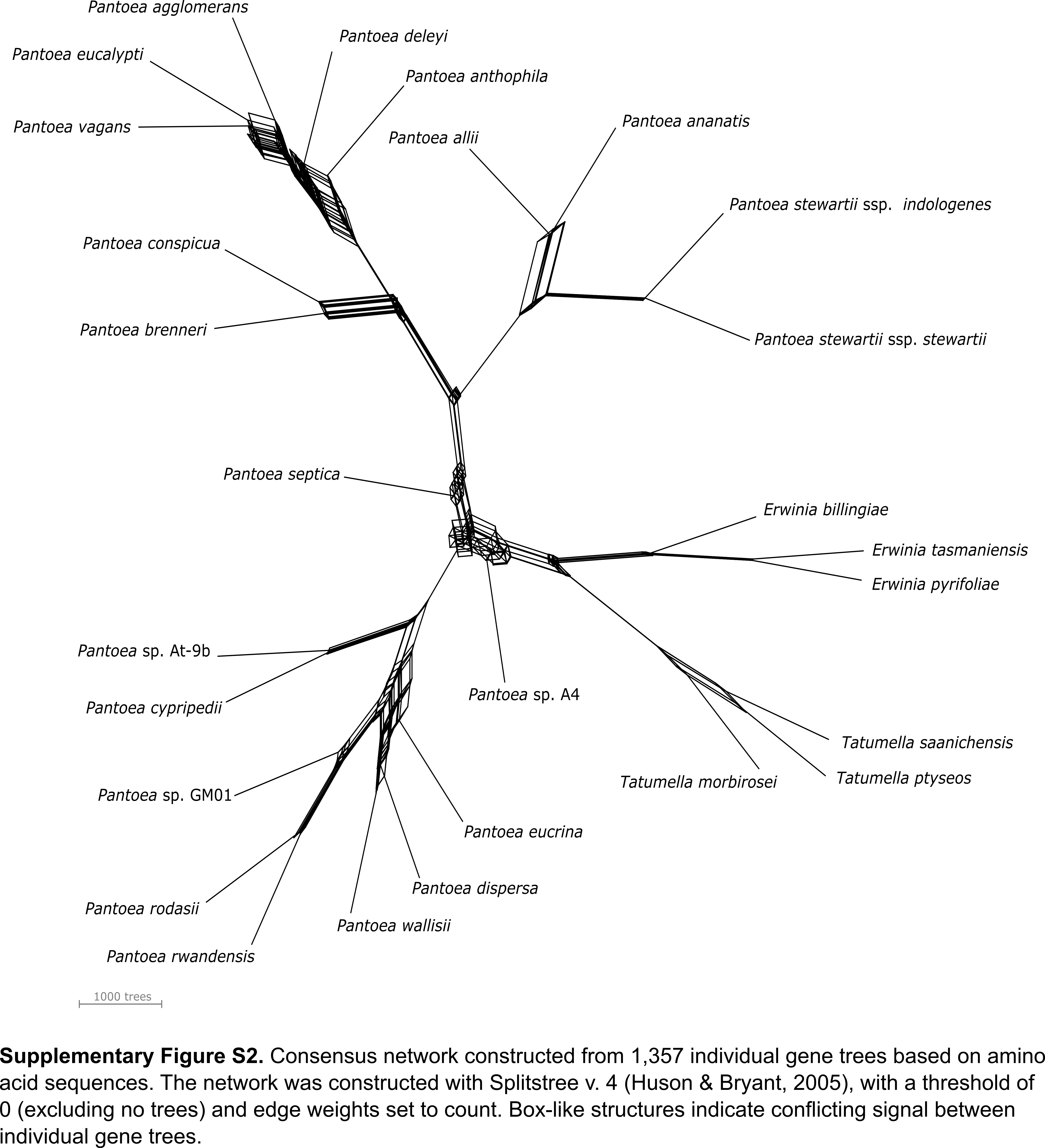

Supplement: Figure S2 [file peerj-07-6698-s002.png]

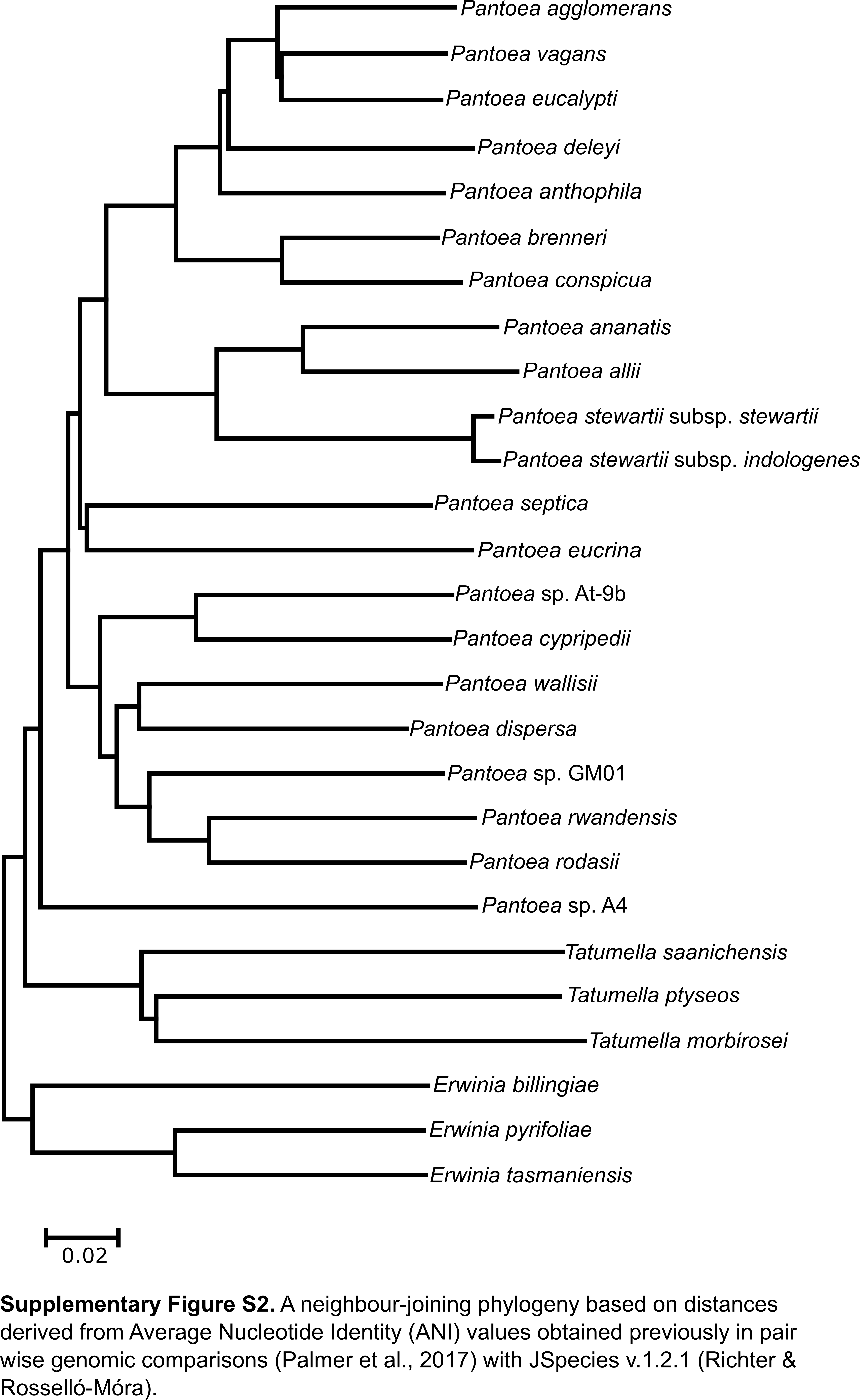

Supplement: Figure S3 [file peerj-07-6698-s003.png]

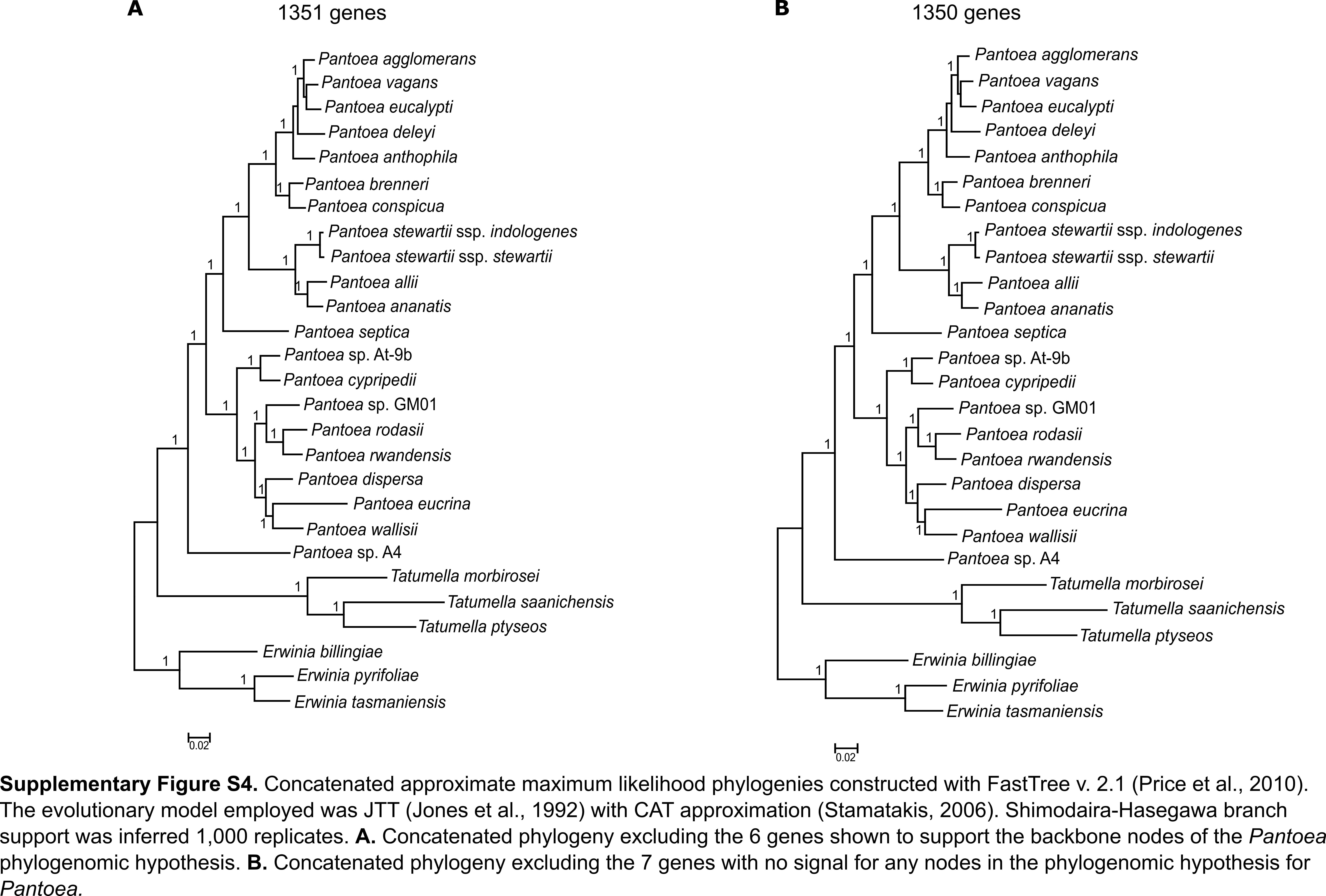

Supplement: Figure S4 [file peerj-07-6698-s004.png]

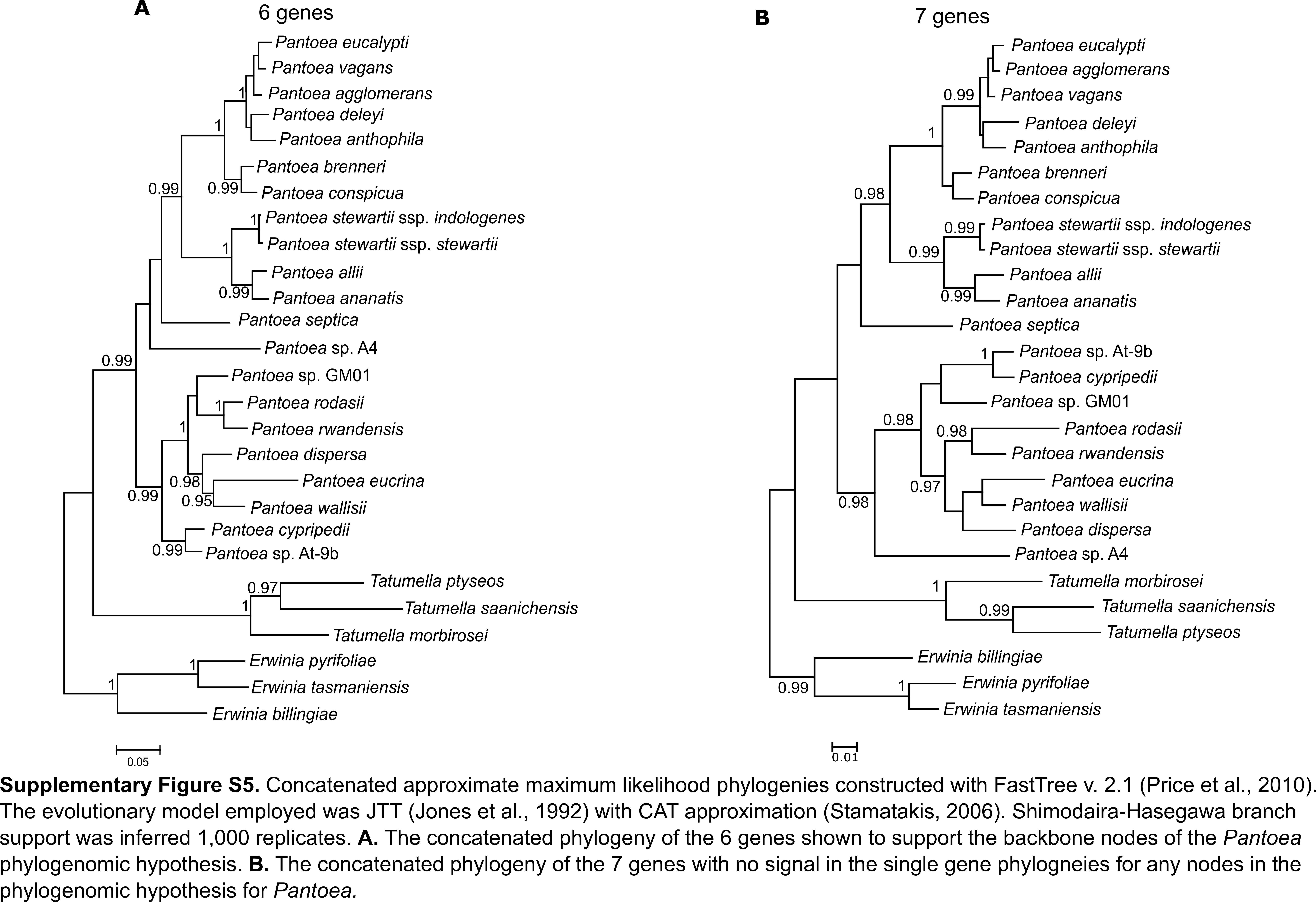

Supplement: Figure S5 [file peerj-07-6698-s005.png]

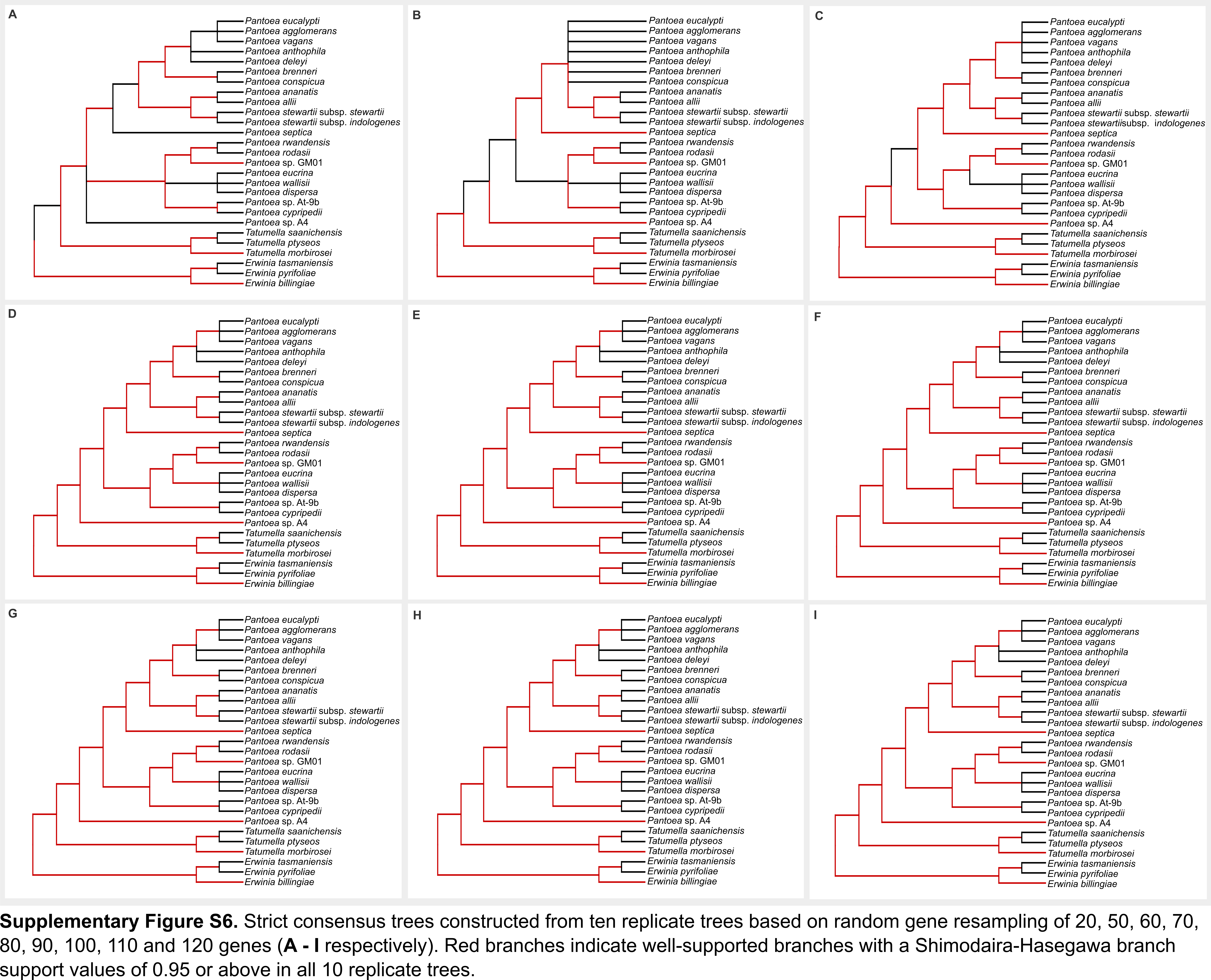

Supplement: Figure S6 [file peerj-07-6698-s006.png]
